# Path analysis of the efficiency in the bud-flower-fruit transition of pitahaya: the biochar-*Trichoderma viride* combination improves yield in *Hylocereus costaricensis* and *H. undatus* cv. Vietnam White

**DOI:** 10.3389/fpls.2026.1901632

**Published:** 2026-07-31

**Authors:** Joseph Campos-Ruiz, Uriel Aldava-Pardave, Jackson Jiménez-Díaz, Nilton Hermoza, Azucena Chávez-Collantes, Cledy Ureta-Sierra, Richard Solórzano

**Affiliations:** 1 Dirección de Servicios Estratégicos Agrarios, Centro Experimental Yanayacu, Instituto Nacional de Innovación Agraria (INIA), Cajamarca, Peru; 2 Dirección de Servicios Estratégicos Agrarios, Estación Experimental Agraria Pucallpa, Instituto Nacional de Innovación Agraria (INIA), Pucallpa, Ucayali, Peru; 3 Dirección de Servicios Estratégicos Agrarios, Centro Experimental La Molina, Instituto Nacional de Innovación Agraria (INIA), Lima, Peru; 4 Dirección de Servicios Estratégicos Agrarios, Estación Experimental Agraria Baños del Inca, Instituto Nacional de Innovación Agraria (INIA), Cajamarca, Peru; 5 Facultad de Ciencias Ambientales, Universidad Científica del Sur (UCSUR), Lima, Peru

**Keywords:** biostimulants, multivariate analysis, plant growth-promoting fungi, reproductive phenology, soil amendment, sustainable agriculture

## Abstract

**Introduction:**

Pitahaya (*Hylocereus* spp.) is an emerging fruit crop, yet the physiological mechanisms by which bioinputs enhance reproductive efficiency and yield remain poorly understood. This study evaluated the effects of rice husk biochar combined with *Trichoderma* viride on reproductive development and fruit production in two pitahaya genotypes.

**Methods:**

A field experiment was conducted in Jaén, Peru, using a randomized complete block design with four treatments (control, biochar, *T. viride*, and biochar + *T. viride*). Reproductive traits and fruit yield were monitored over two production cycles and analyzed using generalized linear models and path analysis.

**Results:**

The combined biochar + *T. viride* treatment enhanced reproductive performance in a genotype-dependent manner. In *H. costaricensis*, it significantly increased flower production and fruit yield, while path analysis identified flowering as the key mediator of yield and revealed a reproductive carrying-capacity threshold of approximately 17 flowers. In contrast, *H. undatus* cv. Vietnam White showed only marginal responses to bioinput application.

**Discussion:**

These findings demonstrate that the effectiveness of biochar and *T. viride* depends on genotype-specific reproductive strategies. The combined bioinput treatment substantially improved reproductive efficiency and yield in *H. costaricensis*, whereas the limited response of *H. undatus* suggests lower phenotypic plasticity and distinct patterns of resource allocation. By integrating longitudinal field evaluations with path analysis, this study provides a physiological framework for precision bioinput management based on reproductive carrying capacity and genotype-specific crop responses, offering new insights for sustainable pitahaya production.

## 1 Introduction

Pitahaya (*Hylocereus* spp.) is a hemiepiphytic cactus native to tropical and subtropical forests of Central America that has gained considerable global importance over the past decades due to the increasing demand for its fruit (Ortiz and Carrillo, 2012). Among the cultivated species, *Hylocereus costaricensis* is native to the dry forests of Nicaragua and Costa Rica, whereas *Hylocereus undatus* is naturally distributed throughout southern Mexico (Verona et al., 2020). Vietnam and China are the world’s leading producer (Tobar and Usiña, 2025), with annual productions of approximately 1.6 and 1.2 million tons, respectively (Zang, 2025; Vinkh, 2026), followed by Thailand, the Philippines, Sri Lanka, and several Latin American countries, including Mexico, Colombia, Costa Rica, Ecuador, and Brazil (Espinoza and Luzuriaga, 2025). In Peru, commercial cultivation has expanded rapidly in regions such as Cajamarca, San Martín, and Amazonas, where favorable edaphoclimatic conditions promote crop establishment and productivity. The growing economic importance of pitahaya is associated not only with its attractive sensory characteristics but also with its high nutraceutical value. The fruit contains abundant bioactive compounds, including betalains, phenolic acids, polysaccharides, oligosaccharides, and essential fatty acids, which exhibit antioxidant, anti-inflammatory, antimicrobial, and other pharmacological activities that contribute to the prevention and management of chronic diseases (Angonese et al., 2021; Jácome et al., 2023; Trindade et al., 2023; Balaji et al., 2025; Mondol et al., 2025; Nagaraj et al., 2026). In Peru, pitahaya cultivation has gained relevance in regions such as Cajamarca, San Martín and Amazonas, where edaphoclimatic conditions of the high jungles favor its development. However, crop productivity faces challenges related to reproductive efficiency (bud drop, pollination failure, or fruit abortion), which represents a primary determinant of final crop yield (Kakade et al., 2022; Santos et al., 2024; Tuesta et al., 2025). Addressing this low efficiency during the bud-to-fruit transition requires the implementation of sustainable agronomic practices grounded in soil and microbial biostimulation.

Fruit production in pitahaya is determined by the successful completion of a hierarchical sequence of reproductive events that includes floral bud initiation, flower development, pollination, fruit set, and fruit growth (Shah et al., 2023; Kakade et al., 2025). In *H. undatus* and *H. costaricensis*, these developmental stages are tightly regulated by environmental conditions, particularly photoperiod and crassulacean acid metabolism (CAM), which synchronize flowering and reproductive development (Nerd et al., 2002a; Le Bellec et al., 2006). However, reproductive success differs markedly between species because *H. undatus* is generally self-compatible, whereas *H. costaricensis* frequently exhibits self-incompatibility, making successful cross-pollination essential for fruit production (Nerd et al., 2002a). Several studies have demonstrated that reproductive losses occur throughout this developmental pathway, as a considerable proportion of floral buds fail to reach anthesis and many flowers do not successfully develop into fruits (Castillo et al., 2005). Flower bud abscission has been associated with intense competition for assimilates during overlapping flowering wave (Chiamolera et al., 2023), whereas irregular climatic fluctuations can disrupt reproductive phenology and reduce pollination efficiency (Chu and Chang, 2020). In addition, self-incompatibility, insufficient pollinator activity, and physiological limitations related to carbon allocation and nutrient availability further restrict fruit set (Silva et al., 2015; Muniz et al., 2019; Chu and Chang, 2020; Cho and Ding, 2021; Kavinmukil et al., 2022; Jadhav et al., 2025). Consequently, the efficiency with which plants convert floral buds into flowers and flowers into fruits ultimately determines crop productivity. Although numerous agronomic studies have evaluated flowering intensity, fruit set, or yield independently, these reproductive stages constitute an integrated biological continuum rather than isolated events (Doke et al., 2024; Trindade et al., 2026). Therefore, analyzing each yield component separately may overlook the causal interactions that govern reproductive success. Identifying where reproductive bottlenecks occur within the bud–flower–fruit transition is essential for developing management strategies that improve yield while maintaining sustainable production systems.

The use of bioinputs has emerged as a sustainable strategy for enhancing crop productivity while reducing dependence on synthetic fertilizers (Chen et al., 2022; Santos et al., 2023; Solórzano et al., 2026). These biological tools optimize the uptake and assimilation of both primary macronutrients—nitrogen, phosphorus, and potassium—and secondary macronutrients, such as calcium, magnesium, and sulfur, alongside essential micronutrients thereby promoting balanced plant nutrition and soil health (Rouphael et al., 2020; Rami et al., 2021). Biochar, a carbon-rich material produced through biomass pyrolysis, has attracted considerable attention because it improves soil structure, increases water-holding capacity, enhances cation exchange capacity, promotes nutrient retention, and stimulates beneficial microbial communities (Lehmann et al., 2011; Escalante et al., 2016; Jatav et al., 2017; Elkhlifi et al., 2023; Kuryntseva et al., 2023; Li and Tasnady, 2023; Nepal et al., 2023). These effects improve nutrient availability, increase nutrient-use efficiency (Ahmed et al., 2025), and create favorable microsites for microbial colonization (Bai et al., 2025), although their magnitude depends on biochar feedstock, pyrolysis conditions, soil characteristics, and application rate (Domingues et al., 2017; Tomczyk et al., 2020; Wu et al., 2021). Recent field studies have further demonstrated that biochar enhances soil carbon sequestration, enzymatic activity, microbial diversity, and crop productivity (Jeong et al., 2026). Rice husk biochar, in particular, represents an attractive strategy for tropical agriculture because it simultaneously recycles an abundant agro-industrial residue and improves soil quality (Pérez et al., 2021; Akhter, 2023; Batool et al., 2023). Likewise, fungi of the genus *Trichoderma* are widely recognized as multifunctional plant-beneficial microorganisms owing to their ability to promote nutrient acquisition, synthesize phytohormones, stimulate root development, induce systemic resistance, and enhance plant tolerance to abiotic stress (Estrada et al., 2019; Sood et al., 2020; Andrzejak and Janowska, 2022; Villavicencio et al., 2025). Among them, *Trichoderma viride* has demonstrated consistent positive effects on plant growth and reproductive development in several agricultural crops through the production of auxins (IAA), gibberellins (GA), cytokinins (CK), and other signaling molecules that regulate plant growth and defense responses (Ousley et al., 1994; Fitrianingsih et al., 2019; He et al., 2020; Illescas et al., 2021; Saldaña et al., 2023).

The simultaneous application of biochar and *T. viride* offers an attractive biological strategy because both components may act synergistically within the rhizosphere. Biochar provides a porous habitat that protects fungal propagules and facilitates long-term colonization, whereas *Trichoderma* actively modifies the rhizosphere through nutrient mobilization, phytohormone production, and interactions with native microbial communities (Sani et al., 2020; Ahmad et al., 2024; de Medeiros et al., 2024; Ullah et al., 2025; Anandan et al., 2026). This complementary interaction has been associated with improvements in soil enzymatic activity, nutrient cycling, rhizosphere metabolism, and crop productivity in cereals and horticultural species (Sani et al., 2020; Cao et al., 2022; Cui et al., 2026; Zhou et al., 2026). Nevertheless, whether this synergistic interaction enhances reproductive efficiency in perennial fruit crops such as pitahaya remains unknown.

Despite the increasing adoption of microbial bioinputs in sustainable agriculture, little is known about the mechanisms through which these technologies influence the sequential transitions from floral buds to flowers and from flowers to fruits in pitahaya. Most previous studies have relied on conventional univariate analyses, which evaluate yield components independently and therefore fail to capture the hierarchical and interdependent relationships among reproductive traits (Iqbal et al., 2024). Because yield is the cumulative outcome of successive developmental transitions, understanding the direct and indirect effects linking floral buds, flowers, fruits, and final fruit yield requires analytical approaches capable of modeling complex causal relationships. Path analysis provides an appropriate framework for quantifying these causal relationships by simultaneously estimating direct and indirect effects among reproductive variables (Valenzuela and Bachmann, 2017). Applied to pitahaya, this approach allows the identification of critical reproductive bottlenecks and the evaluation of whether biological soil amendments modify the efficiency of the bud–flower–fruit transition. In this study, these potential mediating variables correspond primarily to the number of flowers (NFL) and the number of fruits (NFR), since they are part of the reproductive transition from flower buds (NFB) to final fruit weight (FRW); this transition could be modulated in particular by the application of biochar and Trichoderma viride.

Based on this rationale, we hypothesized that (i) the combined application of rice husk biochar and *Trichoderma viride* enhances the efficiency of the floral bud–flower–fruit transition by improving the plant’s physiological carrying capacity, and (ii) this biostimulatory effect exhibits cultivar-dependent nonlinear saturation patterns because of the contrasting reproductive biology of *H. costaricensis* and *H. undatus* cv. Vietnam White. Therefore, the objective of this study was to evaluate the effects of the combined application of rice husk biochar and *T. viride* on the efficiency of the floral bud–flower–fruit transition and its contribution to fruit yield in two pitahaya cultivars using path analysis to identify the direct and indirect mechanisms underlying cultivar-specific responses.

## 2 Materials and methods

### 2.1 Study area

**Figure 1:**
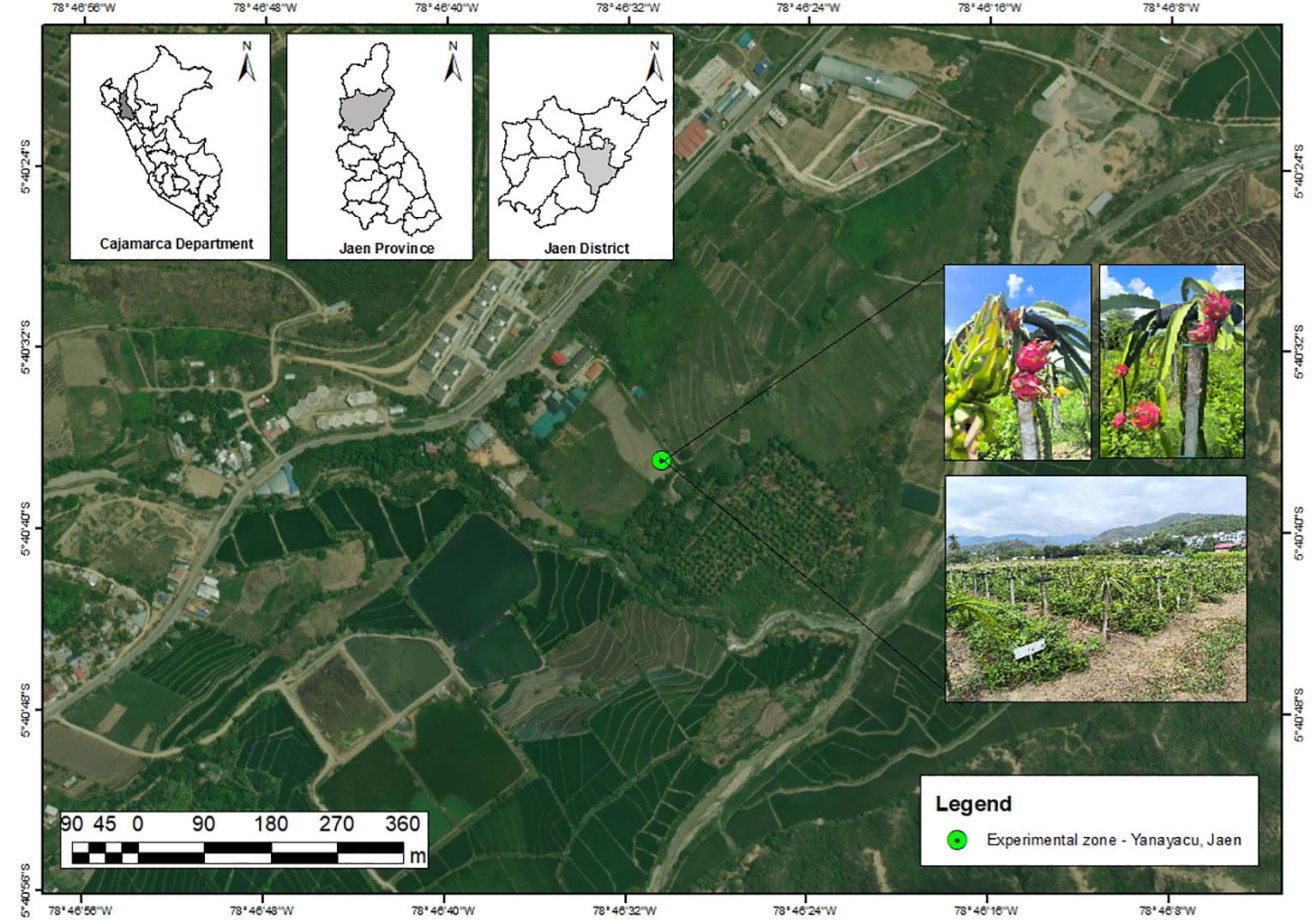
Experimental site location map.

The study was conducted under field conditions at the Yanayacu Experimental Center of the National Institute of Agrarian Innovation (INIA), located in the district and province of Jaén, Cajamarca region, Peru. The experimental plot was established at 5° 40′ 30″ S and 78° 47′ 50″ W; at 658 ma.s.l. (**Figure 1**). The study site is located within the middle basin of the Marañón River, an area characterized by undulating and rugged terrain, inter-Andean valleys, fluvial terraces, and hillslopes with moderate to steep gradients (Poma and Alcantara, 2011). With a photoperiod of 12 hours of light and 12 hours of darkness. The study area has a mean annual temperature ranging from 29 to 33 °C and a mean annual precipitation ranging from 900 mm and 1200 mm (SENAMHI, 2020). This marked seasonality influences soil moisture dynamics throughout the year.

Monthly average meteorological records were obtained from the Jaén weather station, located near the experimental plot and integrated into the SENAMHI, under the administration of the Regional Government of Jaén (**Supplementary Figure 1**).

Initial soil properties (0–20 cm, LABSAF Yanayacu Laboratory): sandy clay loam texture (48.7% sand, 30.1% clay, 21.3% silt); pH 7.6; EC 13 mS m^+^¹; OM 2.1%; total N 1.1 mg g^+^¹; available P 8.2 mg kg^+^¹; available K 109 mg kg^+^¹; CaCO_3_ content 3.0%; CEC 18.2 c mol kg^+^¹. Based on the available soil information and considering the absence of a detailed description of diagnostic horizons, the soil was provisionally classified as an Inceptisol according to the USDA Soil Taxonomy (International Organization for Standardization (ISO), 1995) and as a Cambisol according to the World Reference Base for Soil (WRB) classification system. These soil groups are typically characterized as young to moderately developed soils and are commonly found in mountainous agricultural landscapes of the Peruvian Andes (USS Working Group WRB, 2015).

### 2.2 Plant material and field establishment

**Figure 2:**
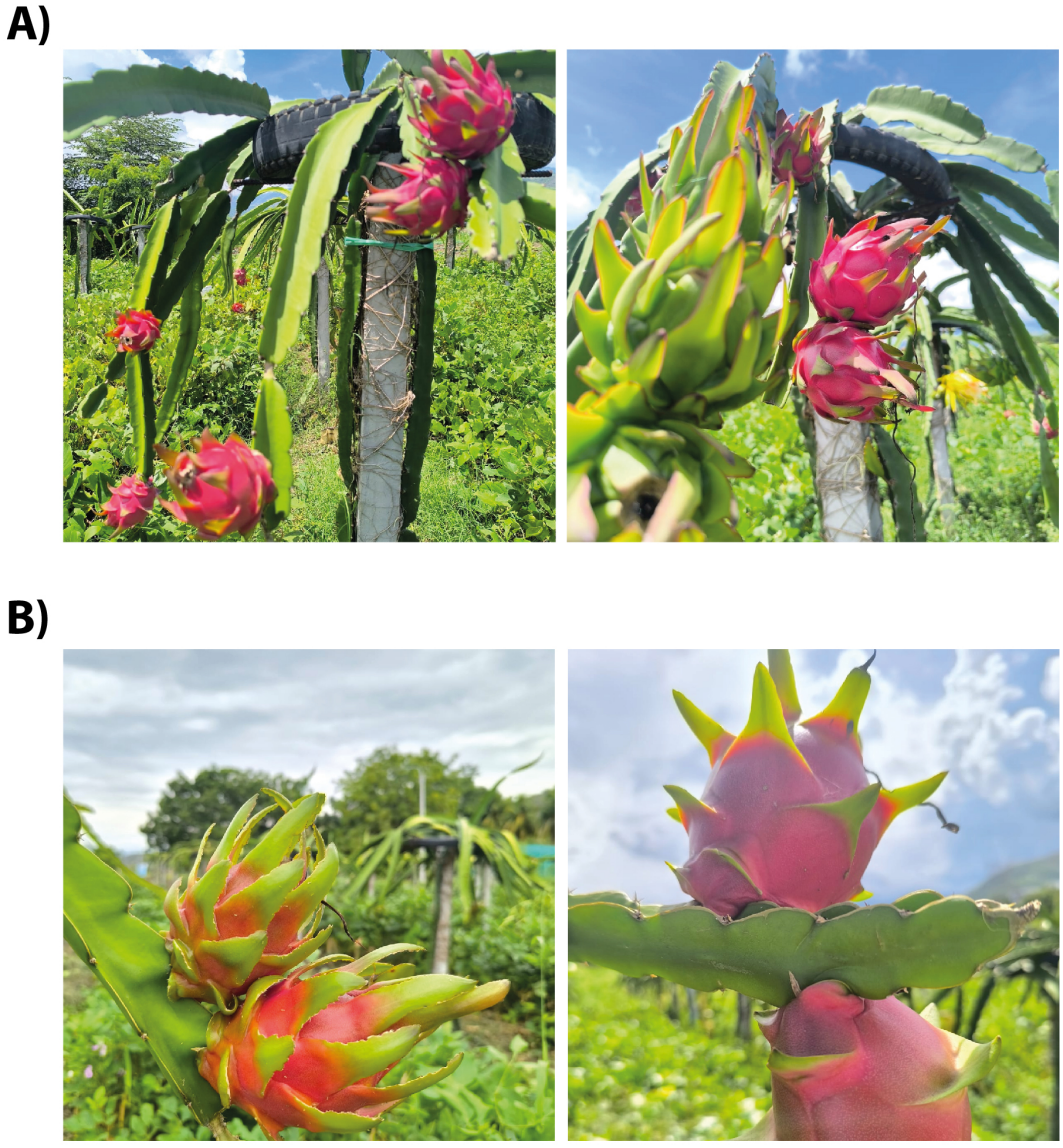
Evaluation of pitahaya fruits: **(A)** *H. costaricensis* and **(B)** *H. undatus* cv. Vietnam white.

Root cuttings (30 cm in length) of *H. costaricensis* and *H. undatus* cv. Vietnam White were obtained from productive commercial plantations owned by Pitahaya Perú SAC (Ancón, Lima, Perú) (**Figure 2**). The cuttings were transplanted into permanent field plots in planting holes measuring 20 x 20 cm, positioned 15 cm from concrete posts 2.0 m in height (0.5 m buried underground) which served as training sorts (Karunakaran et al., 2026) (**Supplementary Figure 2**). Each post sorted two cuttings, with a spacing of 2.5 m between posts and 3.0 m between rows (1600 plants ha^+^¹). The posts provided structural sort during the development of secondary branches (cladodes) and canopy formation.

*H. costaricensis* is a fast-growing cactus species capable of producing its first fruits within the first year after establishment, with subsequent fruiting cycles occurring over approximately five months each year (Sayeri et al., 2026). *H. undatus* cv. Vietnam is recognized for its high fruit quality and commercial value; however, information regarding its phenological development remains limited (Kishore, 2016). Fruits of *H. undatus* cv. Vietnam White are characterized by a red peel and white flesh, whereas the distinctive feature of *H. costaricensis* is its red peel combined with red flesh (Wybraniec et al., 2007).

Both species possess a succulent, triangular stem with undulating ribs bearing areoles and spines. The areoles are located along the ribs and serve as the sites of origin for both spines and floral buds. Areoles and spines exhibit considerable variation in shape, color, and appearance, and are regarded as important morphological descriptors for species characterization. The onset of the reproductive phase is marked by the emergence of cream-colored, spherical floral buds from the areoles. The development of a floral bud into a fully open flower requires approximately 25–35 days. Pitahaya is a photoperiod-sensitive crop, according to the data, it requires between 12 to 14 hours of sunlight per day (Karunakaran et al., 2026), with flowering induced under long-day conditions. In tropical climates, plants may undergo up to 4–6 flowering cycles per year (Jiang et al., 2012).

In this study, *H.s costaricensis* was characterized by an early reproductive habit, producing fruits before reaching 1 year of age. Consequently, this pattern enabled the evaluation of two clearly defined production cycles over a period of nearly two years. In contrast, *H. undatus* cv. Vietnam White, displayed delayed reproductive development under the conditions of the study area, limiting the evaluation to a single production cycle.

### 2.3 Experimental design and treatments

Two pitahaya species (*H. costaricensis* and *H. undatus* cv. Vietnam White) were evaluated in a randomized complete block design with three replicates and four factorial treatments (biochar × TV), totaling 12 experimental units. Each experimental unit consisted of a plot containing six plants of *H. costaricensis* and six of *H. undatus* cv. Vietnam White (12 plants plot^+^¹; 144 total plants). Within each plot, measurements were recorded at the individual plant level and treated as subsamples (non-independent observations) nested within the experimental unit. The factors evaluated were rice husk biochar (absence or presence) and *T. viride* (absence or presence) (**Table 1**). The planting density was 1600 plants ha^+^¹, calculated based on a spacing of 2.5 m × 2.5 m.

**Table 1:** Treatments applied in pitahaya (*H. costaricensis* and *H. undatus* cv. Vietnam White).

N	Biochar	*Trichoderma viride*	Treatments	Repetitions
1	–	–	T01	3
2	+	–	T02	3
3	–	+	T03	3
4	+	+	T04	3

The table summarizes the treatment combinations derived from the factorial arrangement of rice husk biochar application and TV inoculation. Factor levels were defined as: without biochar (–), with biochar (+), without Trichoderma viride (–), and with Trichoderma viride (+).

### 2.4 Biochar

Rice husk biochar was produced from rice husk collected from local rice milling facilities through slow pyrolysis at 710 °C for 55 min under strictly oxygen-limited conditions, following the protocol described by (El-Naggar et al., 2019). Pyrolysis was performed in a *top-lit updraft* (TLUD) gasifier, an upward-draft reactor with top ignition that enables controlled biomass carbonization under limited oxygen sly, thereby producing a stable biochar with a high carbon content (Cornelissen et al., 2016). Physicochemical characterization was performed at the Soil, Water and Foliar Analysis Laboratory Network of the National Institute of Agrarian Innovation (LABSAF-INIA). The pH and electrical conductivity (EC) were determined according to Test Methods for the Examination of Composting and Compost (TMECC) (International Biochar Initiative (IBI), 2015). Organic carbon content was measured following ISO 10694:1995 (International Organization for Standardization (ISO), 1995); whereas heavy metal concentrations were quantified using EPA 3051A. Detailed physicochemical properties of the biochar are provided **Supplementary Table 1**.

### 2.5 Preparation and application of the amendment

Rice husk biochar was inoculated with TRICHOMAX^®^ (TV, 1 × 10¹² conidia kg^+^¹; SOLAGRO) at a rate of 5 g per 500 mL, resulting in a final concentration of 10^6^ conidia mL^+^¹, adapted from the SOLAGRO technical data sheet. The inoculated biochar was prepared one week before planting. T04 received 500 mL plant^+^¹ of inoculated biochar, equivalent to the biochar dose applied in T02 (500 mL plant^+^¹ = 312 kg ha^+^¹), ensuring direct comparison between treatments. T03 consisted solely of TRICHOMAX^®^ (5 g plant^+^¹ = 8 kg ha^+^¹) adapted from (Cui et al., 2026). Applications were carried out twice: i) one week before planting; ii) four months after establishment (10 cm from the root collar and coinciding with the emergence of actively growing secondary shoots) adapted from (Zhou et al., 2026).

### 2.6 Crop agronomic management

All plots received uniform agronomic management throughout the experiment. Drip irrigation was applied every other day (2.3 L plant^+^¹ months 1-3; 9 L plant^+^¹ from month 4 until the end of the production cycle) and liquid fertilization was applied every two months (urea 46% N: 0.47 kg; diammonium phosphate 46-0-0: 0.57 kg; KCl 60% K_2_O: 0.68 kg dissolved in 200 L of water. The solution was applied at a rate of 1.3 L plant^+^¹, 10 cm from the root collar; NPK 120-60–120 kg ha^+^¹ year^+^¹) according to local technical recommendations for pitahaya cultivation. Uniform baseline management was implemented to ensure that any observed differences among treatments could be attributed to experimental factors rather than to variation in water or nutrient availability.

### 2.7 Evaluated variables

#### 2.7.1 Growth and vegetative development variables

Plant height was assessed weekly in both pitahaya species using a 100 cm measuring tape. In *Hylocereus costaricenses*, measurements were recorded from March to October, whereas in *H. undatus* cv. Vietnam White evaluations were conducted from March to November 2024.

#### 2.7.2 Reproductive variables

Reproductive variables (VR) included the number of floral buds (NFB), number of flowers (NFL), number of fruits (NFR) and fruit weight (FRW) as described in **Table 2**.

**Table 2:** Evaluation of reproductive variables.

Variety	RV	Season 1						Season 2			
		2024		2025				2025		2026	
		Nov	Dec	Jan	Feb	Mar	Apr	Nov	Dec	Jan	Feb
*Hylocereus costaricensis*	NFB	–	M01	M02	M03	–	–	M04	–	M05	–
	NFL	–	M01	M02	M03	–	–	M04	–	M05	–
	NFR	–	M01	M02	M03	–	–	–	M04	M05	–
	FRW	–	–	M01	M02	M03	–	–	M04	M05	M06
*Hylocereus undatus* cv. Vietnam White	NFB	–	–	–	–	–	–	M01	–	M02	–
	NFL	–	–	–	–	–	–	M01	–	M02	–
	NFR	–	–	–	–	–	–	–	M01	M02	–
	FRW	–	–	–	–	–	–	–	M01	M02	M03

**(**–**).** Absence of the reproductive variable.

Green = number of floral buds (NFB); blue = number of flowers (NFL); yellow = number of fruits (NFR); purple = fruit weight (FRW). Color shading is used solely for visual identification of the evaluated reproductive variables and has no statistical or biological meaning.

#### 2.7.3 Yield variables

Yield (plant^+^¹ kg) was determined from the fresh weight of fruits harvested at commercial maturity over a three-month harvest period for each production cycle. The first production cycle was evaluated from January to March 2025, and the second production cycle from December 2025 to February 2026.

### 2.8 Statistical analysis

Statistical analyses were performed in R version 4.5.0 (R Core Team, 2025) through generalized linear models (GLMs) fitted with the *glmm*TMB package. For each response variable, alternative distributions were evaluated according to the nature of the data, and dispersion was modeled as a function of covariates when necessary. Model selection was based on the Akaike Information Criterion (AIC) using the *MuMIn* package, because the AIC allows for the comparison of alternative candidate models while simultaneously considering both model fit and complexity, which favors the selection of the most parsimonious model (Akaike, 1974; Barton, 2009). The selected models, including their distributions and link functions, are presented in **Supplementary Table 2**, whereas cases in which dispersion was explicitly modeled are detailed in **Supplementary Table 3**. Model validation was conducted using simulated residuals generated with the *DHARMa* assessing residual uniformity, outlier occurrence, dispersion, and homoscedasticity. Subsequently, estimated marginal means were calculated for each treatment using the emmeans package, and multiple comparisons were performed using a false discovery rate (FDR) correction. The significance level was set at 0.05, as this threshold provides an accepted balance between Type I error control and statistical power (Haile, 2023). Finally, reproductive path models were fitted separately for each pitahaya variety and production cycle using the *lavaan* package. Model fit was evaluated using the chi-square test (χ²), Comparative Fit Index (CFI), Tucker-Lewis Index (TLI), Root Mean Square Error of Approximation (RMSEA), and Standardized Root Mean Square Residual (SRMR). Direct, indirect, and total effects were estimated whenever applicable.

## 3 Results

### 3.1 Hylocereus costaricensis

The fixed component of the GLMMs revealed a strong effect of evaluation time on all reproductive traits (p < 0.01), identifying it as the primary source of variation. Biochar application also exerted a significant influence (p < 0.05) on most variables, although its effect on stem length (SL) was marginal (p= 0.06). TV significantly affected the NFL, NFR and FRW. The two-way interactions exhibited trait-specific responses: TI: BI and TI: TV influenced FRW, whereas BI: TV affected NFL, NFR and FRW. In contrast, the three-way interaction TI: BI: TV showed a significant effect only on NFB (**Table 3**).

For both FRW and NFR, the first evaluation was excluded from the analysis due to the predominance of zero values. Subsequently, a hurdle model was fitted for FRW. **Table 3** presents the results of the positive component, which corresponds to fruit weight when production occurred (values greater than zero). For the binary component, representing the absence or presence of production, time did not exert a significant effect (p > 0.01). Nevertheless, a marginal increase in the probability of production was observed, rising from 0.38 in the second evaluation to 0.44 in the third evaluation and reaching 1.00 from the fourth evaluation onward. These results indicate that from the fourth evaluation onward. The results indicate that, from the fourth evaluation onward, all experimental units contained at least one plant producing fruits. This progressive stabilization toward a uniform special distribution of fruit production aligns with the characteristically synchronized, episodic flowering flushes reported for *Hylocereus* species (Nerd et al., 2002b; Le Bellec et al., 2006). In pitahaya cultivation, early-season reproductive flushes often display lower intensity and higher inter-plant asynchrony due to initial microclimatic and resource-allocation transitions, whereas subsequent mid-season peaks establish a highly uniform and reliable fruit-bearing pattern across experimental plots. Similar temporal dynamics, where fruit-set probability achieves full uniformity only after initial transitional phases, have been widely documented in other tropical and subtropical perennial crops with flush-based fruiting habits, such as mango (*Mangifera indica*) and citrus (*Citrus* spp.) (Goldschmidt, 1999; Ramírez and Davenport, 2010).

**Table 3:** Fixed and random effects on the mean reproductive components of *H. costaricensis*: a longitudinal GLMM analysis.

Factors	NFB	NFL	NFR	FRW	SL
Fix effect (p-value)					
Block	0.30	0.26	0.14	< 0.01	0.03
Time (TI)	< 0.01	< 0.01	< 0.01	< 0.01	< 0.01
Biochar (BI)	0.03	< 0.01	< 0.01	< 0.01	0.06
*Trichoderma viride* (TV)	0.74	0.02	< 0.01	< 0.01	0.38
TI: BI	0.38	0.51	0.90	< 0.01	0.84
TI: TV	0.28	0.09	0.43	< 0.01	0.28
BI: TV	0.14	< 0.01	0.01	< 0.01	0.58
TI: BI: TV	0.01	0.30	0.65	0.33	0.99
Random effect ( $\hat{\sigma }$)					
Plot	0.16	0.15	0.17	0.00	0.02

The table summarizes the significance levels of the fixed effects and the standard deviation of the random effect. The fixed-effects structure included block as a control factor and evaluation time (TI), biochar application (BI), T. viride inoculation (TV), and their interactions as explanatory factors. The random effect was plot, included to account for the dependence among repeated measurements and the variability between plots. The evaluated response variables were the number of floral buds (NFB), number of flowers (NFL), number of fruits (NFR), fruit weight (FRW), and stem length (SL). For FRW, the results presented in the table correspond exclusively to positive values.

**Figure 3:**
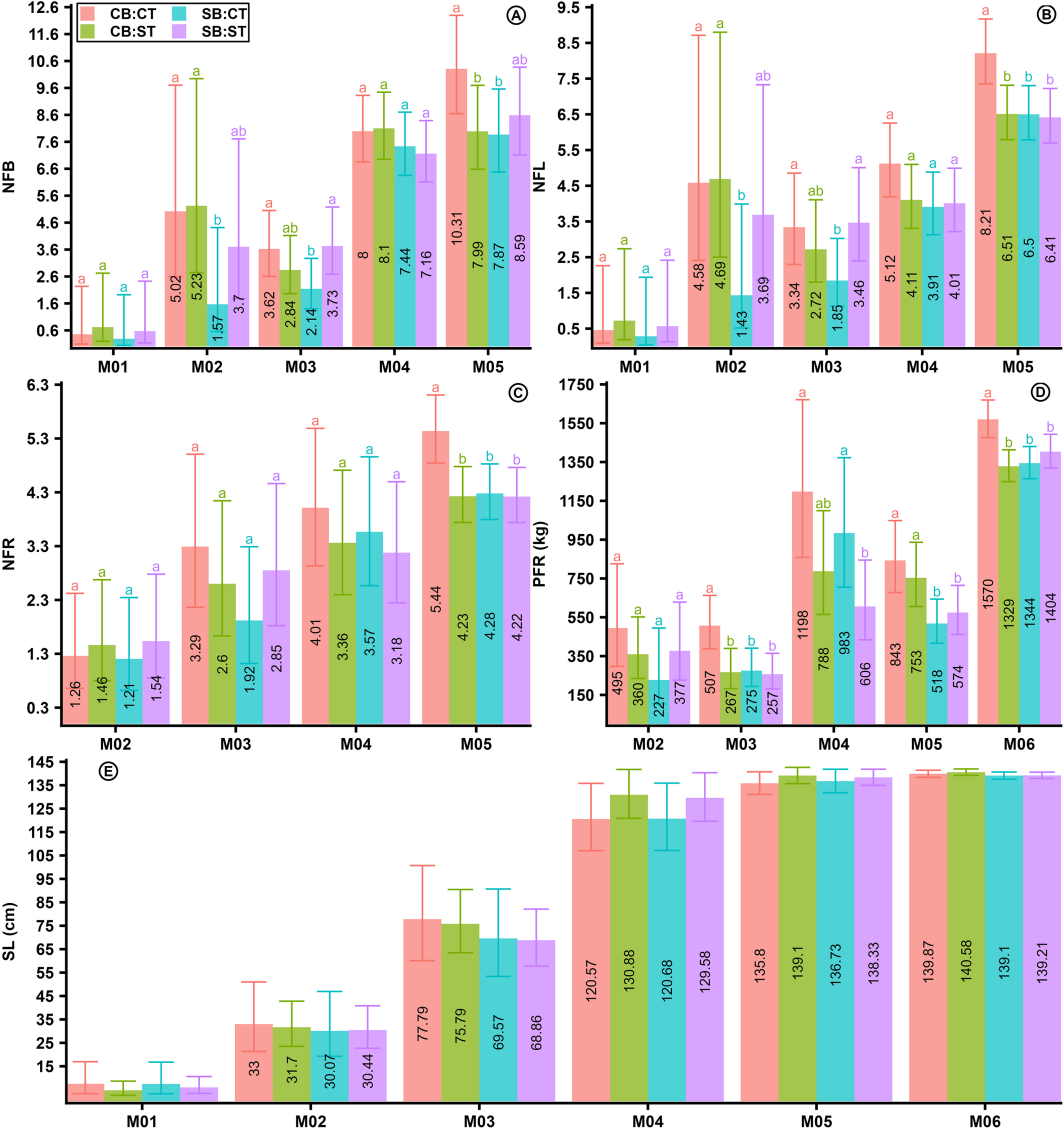
Adjusted means of the reproductive components of *H. costaricensis* across treatments at each evaluation period. **(A–E)** show the adjusted means for the number of floral buds (NFB), number of flowers (NFL), number of fruits (NFR), fruit weight (FRW), and stem length (SL), respectively. Treatments were defined as follows: T01, no biochar + no *T. viride*; T02, biochar + no *T. viride*; T03, no biochar + *T. viride*; and T04, biochar + *T. viride*. Error bars represent 95% confidence intervals. Different letters within each panel and evaluation period indicate significant differences among treatments after false discovery rate (FDR) correction (*P* < 0.05). M01–M06 correspond to the evaluation periods described in **Table 2**. For NFR and FRW, M01 was excluded because of the high frequency of zero values. For FRW, adjusted means were estimated using positive observations only.

The random effects component of the GLMMs revealed substantial heterogeneity among experimental units (plots) for most reproductive traits. However, for FRW, the variance associated with the random effect was close to zero, indicating that plots did not differ markedly with respect to this variable.

the three-way interaction TI x BI x TV was significant only for the number of floral buds, planned multiple comparisons were conducted within each evaluation period for the BI x TV interaction, as one of the objectives of this study was to assess the response to biochar and TV combinations at different stages of reproductive development. This analysis enabled the identification of treatment-specific differences within each evaluation period and provided insight into the temporal dynamics of reproductive component responses (**Figure 3**).

For NFB, similar values were observed among treatments during the first evaluation. However, in the second evaluation, T04 and T02 exhibited higher values than T03, whereas T01 showed an intermediate response reflects the baseline reproductive capacity of the genotype understands localized conditions, which lacks the physiological acceleration provided by the synergistic bioinputs in T04 (Nerd et al., 2002b). During the third evaluation, T04 and T01 were superior to T03, with intermediate values for T02. No significant differences among treatments were detected in the fourth evaluation. In contrast, during the fifth evaluation, T04 achieved the highest NFB, differing mainly from T02 and T03 (**Figure 3A**). For NFL, treatments responses were like NFB, standing out during the fifth evaluation, only T04 exhibited the highest response, differing from all other treatment combinations (**Figure 3B**).

Additionally, for NFR treatment differences were not evident during M02, M03 and M04. However, a clear separation among treatments emerged in M05, where T04 achieved the highest number of fruits, differing from T02, T03 and T01(**Figure 3C**). Regarding FRW, no significant differences among treatments were observed in M02. In M03, T04 showed a greater response than the remaining treatments, whereas in M04, T04 and T03 exhibited the highest values, particularly in comparison with T01. In M05, T04 and T02 outperformed T03 and T01. Finally, in M06, T04 once again achieved the highest fruit weight (**Figure 3D**). This consistent superiority of the combined Biochar + *Trichoderma viride* treatment (T04) in final fruit number and weight is strongly sorted by previous research in cactaceous and tropical perennial species. Dual inoculation of organic matrices with plant growth-promoting fungi (*Trichoderma* spp.) has been shown to exponentially increase root surface area and enhance phosphorus and micronutrient solubilization, which directly translates into elevated net carbon assimilation and efficient photoassimilate partitioning toward reproductive sinks during peak production phases (López et al., 2015; Vinci et al., 2018). No significant differences in stem length were detected among treatments at any of the evaluation periods (**Figure 3E**).

**Figure 4:**
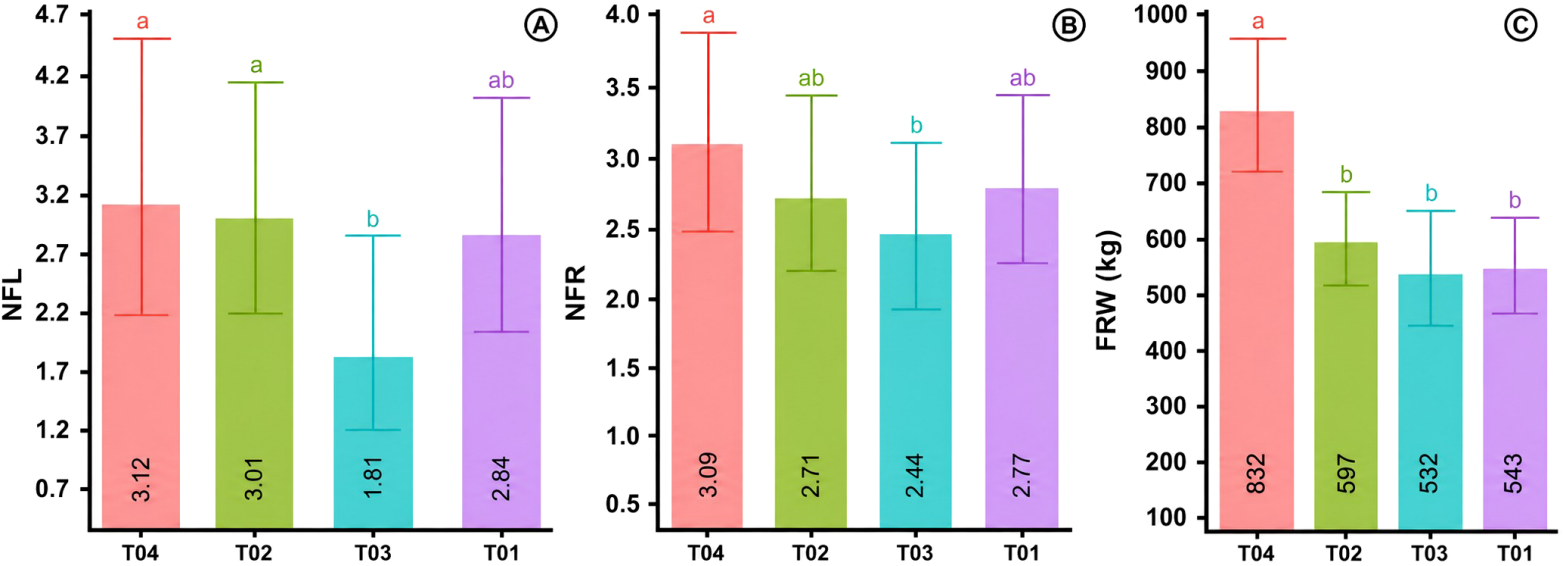
Estimated marginal means of the reproductive components of *H. costaricensis* across treatment combinations. The figure shows the adjusted means for the evaluated treatments. **(A–C)** correspond to the number of flowers (NFL), number of fruits (NFR), and fruit weight (FRW), respectively. Treatments were defined as follows: T01, no biochar + no *T. viride*; T02, biochar + no *T. viride*; T03, no biochar + *T. viride*; and T04, biochar + *T. viride*. Error bars represent 95% confidence intervals. Different letters within each panel indicate significant differences among treatments after false discovery rate (FDR) correction (*P* < 0.05). For FRW, adjusted means were estimated using positive observations only.

In addition to the comparisons performed within each evaluation period, and because the BI x TV interaction was significant for NFL, NFR, and FRW, the estimated marginal means of this interaction were further examined to determine the combined effect of biochar and TV on these reproductive components. This analysis made it possible to assess whether the differences observed at specific evaluation periods were consistently reflected as an overall trend across treatment combinations (**Figure 4**).

For NFL, the T04 and T02 combinations presented the highest values, mainly surpassing T03, while T01 showed an intermediate behavior (**Figure 4A**). In the NFR, although T04 registered the highest average value, the differences between combinations were not statistically significant (**Figure 4B**). In contrast, for FRW, the T04 combination achieved the highest mean value and differed from the remaining treatment combinations, indicating a favorable response when biochar was combined with TV (**Figure 4C**).

Following the evaluation of the temporal dynamics of the biochar x TV interaction at each assessment period, the analysis was complemented with an integrated evaluation across the entire study. This approach considered the cumulative totals of NFB, NFL, NFR, and FRW, as well as SL recorded at the end of the evaluation period, thereby providing an overall assessment of reproductive and productive performance. Under this integrated analysis, biochar exerted significant effects on NFB, NFL, and FRW, together with a marginal effect on SL, whereas *T. viride* significantly affected only FRW. In turn, the BI x TV interaction was significant for NFL and FRW and showed a marginal effect on NFB.

Multiple comparisons associated with the significant and marginal effects revealed that the T03 treatment achieved the highest cumulative values for both NFB and NFL, differing primarily from T03, whereas T01 and T02 exhibited intermediate values. For NFR, although the BI x TV interaction was not significant, a descriptive trend toward higher values was observed in T03. In the case of FRW, T03 produced the highest cumulative value and differed significantly from all other treatment combinations.

### 3.2 *Hylocereus undatus* cv. Vietnam White

In *H. undatus* cv. Vietnam White, the fixed component of the GLMM revealed that evaluation time significantly affected only SL (p < 0.01), with no significant effects detected for NFB, NFL, or NFR. Inoculation with TV had a significant effect exclusively on NFB (p = 0.02), whereas biochar application did not exert significant main effects on any of the evaluated variables. Regarding the two-way interactions, TI: TV and BI: TV were not significant, whereas TI: BI significantly affected only SL (p = 0.04). Finally, the three-way interaction (TI: BI: TV) did not significantly influence any of the response variables analyzed. The random component indicated that between-plot variability was expressed primarily in the reproductive variables NFB, NFL, and NFR, whereas stem length exhibited a more homogeneous response across experimental units (**Table 4**).

**Table 4:** Fixed and random effects on the reproductive components *of H. undatus* cv. Vietnam White: longitudinal analysis using generalized linear mixed models (GLMMs).

Factors	NFB	NFL	NFR	SL
Fix effect (p-value)				
Block	0.18	0.21	0.46	0.61
Time (TI)	0.58	0.77	0.72	< 0.01
Biochar (BI)	0.26	0.35	0.77	0.40
Trichoderma (TV)	0.02	0.21	0.22	0.97
TI: BI	0.59	0.48	0.26	0.04
TI: TV	0.88	0.71	0.95	0.86
BI: TV	0.45	0.81	0.20	0.86
TI: BI: TV	0.55	0.10	0.94	0.88
Random effect ( $\hat{\sigma }$)				
Plot	0.78	0.71	0.93	0.01

The table summarizes the significance levels of the fixed effects and the standard deviation of the random effect. The fixed-effects structure included block as a control factor and evaluation time (TI), biochar application (BI), T. viride inoculation (TV), and their interactions as explanatory factors. Plot was included as a random effect to account for the dependence among repeated measurements and the variability between plots. The evaluated response variables were the number of floral buds (NFB), number of flowers (NFL), number of fruits (NFR), and stem length (SL). Because NFB, NFL, and NFR exhibited a high frequency of single-count observations, these variables were dichotomized before model fitting into two categories (1): presence of a single observed structure and (2) presence of than one observed structure.

**Figure 5:**
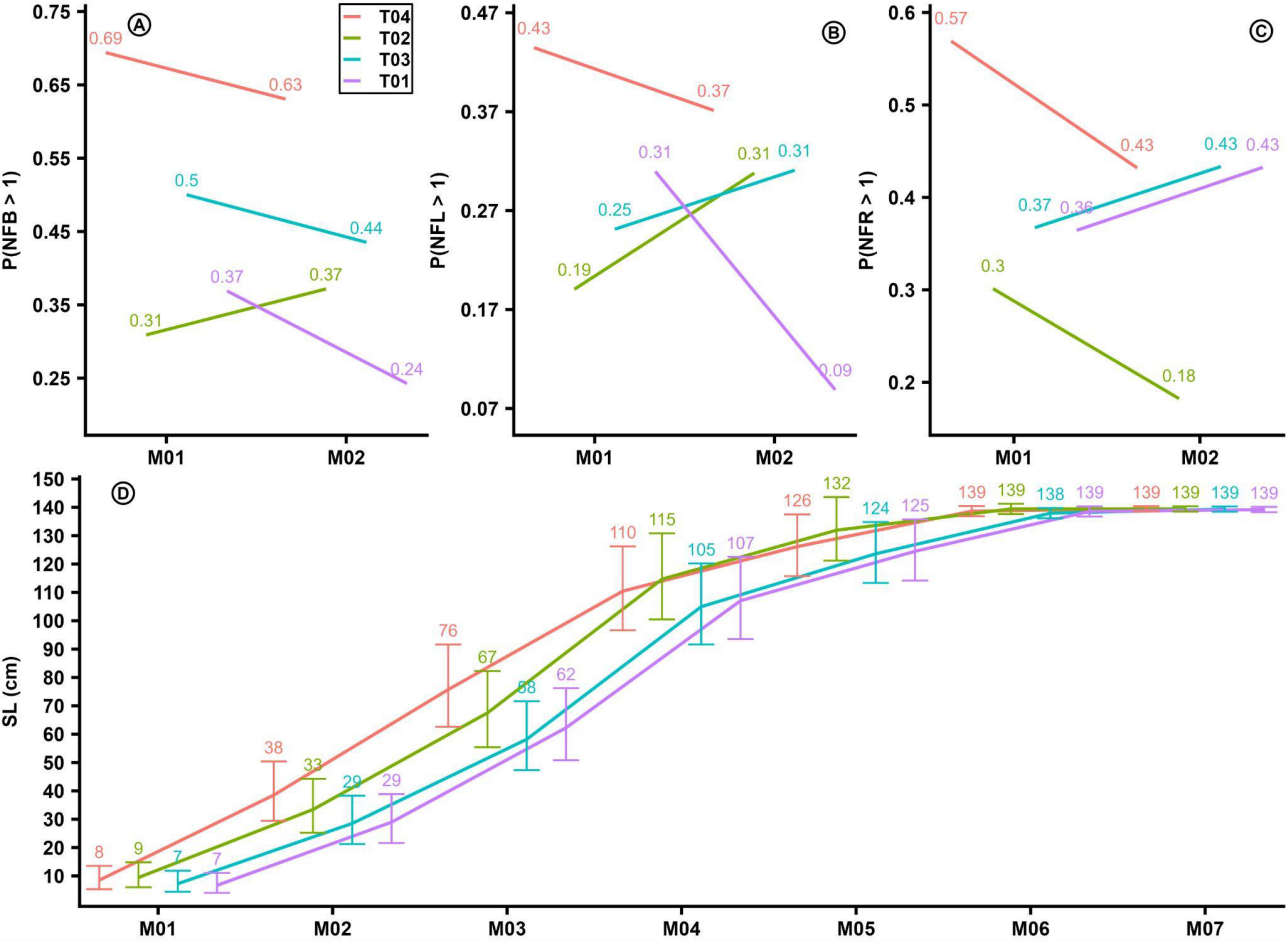
Estimated marginal means of the reproductive components of *H. undatus* cv. Vietnam White across treatments at each evaluation period. **(A–C)** show the predicted probabilities that the number of floral buds (NFB), flowers (NFL), and fruits (NFR), respectively, exceeds one [*P*(count > 1)] at each evaluation period, whereas panel D shows the estimated marginal means of stem length (SL). The lines connect the adjusted estimates across evaluation periods to facilitate visualization of treatment responses over time. Error bars in **(D)** represent 95% confidence intervals. M01–M07 correspond to the cumulative monthly evaluation periods described in **Table 2**. Treatments were defined as follows: T01, no biochar + no *T. viride*; T02, biochar + no *T. viride*; T03, no biochar + *T. viride*; and T04, biochar + *T. viride*.

Given the specific objective of comparing the biochar x TV interaction within each evaluation period, planned comparisons among treatment combinations were performed conditionally on time. Because the three-way interaction (TI: BI: TV) was not significant, these results were not interpreted as evidence of a temporal shift in the interaction effect, but rather as descriptive trends of simple effects within each evaluation period For the reproductive variables, expressed as the probability of observing values greater than one, no significant differences among treatments were detected for NFB (**Figure 5A**), NFL (**Figure 5B**), or NFR (**Figure 5C**) across the evaluation periods, although numerical variation among treatment combinations was observed, with a tendency toward higher values in T04. In contrast, stem length increased progressively from May to November 2025; however, no significant differences among treatments were detected at any individual evaluation period (**Figure 5D**).

Additionally, to evaluate the integrated response of *H. undatus* cv. Vietnam White: through the entire experimental period a complementary analysis was conducted using the cumulative values of NFB, NFL, NFR, and FRW, together with final stem length measured at the end of the evaluation period. This approach provided a comprehensive assessment of the overall productive performance of the treatments. The cumulative analysis was particularly relevant for FRW, a variable that could not be reliably assessed within the temporal framework because of the high frequency of zero values recorded during several evaluation periods.

In the global GLM analysis, TV inoculation maintained a significant effect only on NFB (*p* = 0.01) and showed a marginal effect on SL (*p* = 0.06), whereas neither biochar nor the biochar x *T. viride* interaction significantly affected any of the evaluated variables. Block effects were significant only for SL (*p* = 0.04). Because the BI x TV interaction was not significant, adjusted treatment means were interpreted descriptively. In this context, the T04 treatment exhibited the highest values for NFB, NFL, and NFR. For fruit weight (FRW), a hurdle model was fitted due to the high frequency of zero values. In the binary component, which evaluated the probability of fruit production (absence vs. presence of fruits), no significant differences were detected among treatments (p > 0.05) (**Table 5**). This statistical uniformity in the initial probability of fruit production indicates that the primary induction of the reproductive phase in *Hylocereus* spp. is fundamentally synchronized by regional macroclimatic signals, such as photoperiod and ambient temperature, which affected all experimental plots equally. Consequently, the soil treatments did not alter the environmental likelihood of a plant entering the fruiting stage; instead, they governed the plant’s capacity to accumulate resources once the stage was initiated. This explains why, despite the non-significant binary threshold, a clear gradient appeared in the descriptive and positive components. The estimated probabilities of fruit production were: 0.44 for T01, 0.50 for T02 and 0.67 for both T03 and T04 (**Table 5**). In the positive component, which included only experimental units containing at least one productive plant (fruit weight > 0), the highest FRW values were observed in T03 and T04. This differentiation in the positive component reinforces the premise that while climate dictates *when* production begins (the binary barrier), the combined biochar and *Trichoderma* application (T04) acts as a rhizosphere-driven metabolic buffer, optimizing nutrient uptake and photoassimilate distribution to maximize final sink size and fruit weight, a dual-regulation mechanism widely observed in flush-based perennial crops (Nerd et al., 2002b). Final stem length (SL), in turn, showed a relatively homogeneous response.

**Table 5:** Fixed effects on the mean reproductive components of *H. undatus* cv. Vietnam White: Global GLM analysis.

Factors / Treatments	NFB	NFL	NFR	FRW	SL
ANOVA (p-value)					
Block	0.23	0.19	0.19	0.66	0.04
Biochar (BI)	0.19	0.28	0.28	0.10	0.47
*Trichoderma viride* (TV)	0.01	0.15	0.15	0.81	0.06
BI: TV	0.16	0.75	0.75	0.82	0.60
Means adjusted according to treatment					
T01	2.94b	2.44	2.44	0.44	139.22
T02	2.88b	2.55	2.55	0.50	140.60
T03	3.21ab	2.61	2.61	0.67	139.11
T04	3.94a	2.83	2.83	0.67	139.89

The table presents the significance levels of the fixed effects, with block included as a control factor and biochar application (BI), Trichoderma viride inoculation (TV), and the BI × TV interaction included as explanatory factors. The evaluated response variables were the number of floral buds (NFB), number of flowers (NFL), number of fruits (NFR), fruit weight (FRW), and final stem length (SL). For FRW, the results presented in the table are based exclusively on positive observations. Adjusted means for each treatment are also reported for each response variable. Different lowercase letters within the same column indicate significant differences among treatments after false discovery rate (FDR) correction (P < 0.05). Treatments were defined as follows: T01, no biochar and no T. viride; T02, biochar and no T. viride; T03, no biochar and T. viride; and T04, biochar and T. viride.

### 3.3 Reproductive pathway model

Following the treatment responses characterization through both the temporal and cumulative analyses, the internal structure of the relationships among reproductive components was further examined using path analysis models. This approach enabled the evaluation of how the floral bud–flower–fruit–fruit weight sequence was organized and whether final yield was primarily associated with direct effects or with indirect effects mediated through intermediate reproductive stages. For *Hylocereus costaricensis*, all reproductive stages were included in the path model because the relationships among them were statistically significant. For this species, the analysis was performed separately for each reproductive cycle, as two distinct reproductive seasons were observed during the study period. In contrast, a single path model was fitted for *H. undatus* cv. Vietnam White, corresponding to the only reproductive cycle observed throughout the experiment. This model included only the number of floral buds (NFB), number of flowers (NFL), and number of fruits (NFR), as these were the only variables that exhibited significant relationships within the path model.

#### 3.3.1 *Hylocereus costaricensis*– first production cycle

**Figure 6:**
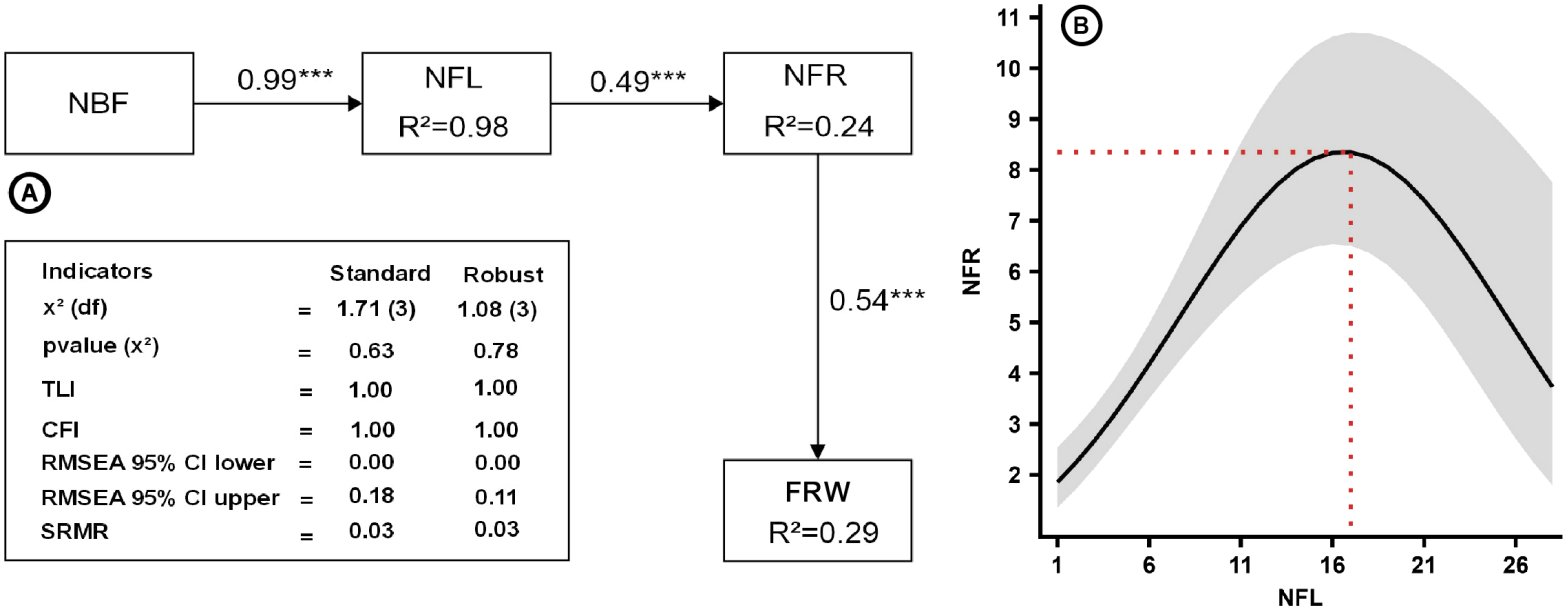
Reproductive path model of *H. costaricensis* during the first production cycle: direct effects and R^2^. **(A)** shows the standardized direct effects, coefficients of determination (R²), and overall goodness-of-fit indices of the path model comprising the number of floral buds (NFB), number of flowers (NFL), number of fruits (NFR), and fruit weight (FRW). **(B)** shows the nonlinear relationship between NFL and NFR. The solid black line represents the fitted model, the shaded area indicates the 95% confidence interval, and the red dashed lines denote the estimated maximum fruit production point. Direct effects are represented by arrows (→), and statistical significance is indicated by *** (*P* < 0.001).

The reproductive path model for the first production cycle showed an adequate fit under robust estimation (χ²(dl) = 1.08 (3), p = 0.78; CFI = 1.00; TLI = 1.00; RMSEA IC 95% [0.00, 0.11]; SRMR = 0.03). The path model included the number of floral buds (NFB), number of flowers (NFL), number of fruits (NFR), and fruit weight (FRW). The estimated model revealed a sequence of significant positive direct pathways linking consecutive stages of reproductive development. Specifically, the number of floral buds had a strong positive effect on the number of flowers (NFB → NFL; β = 0.99), the number of flowers positively affected the number of fruits (NFL → NFR; β = 0.49), and the number of fruits positively influenced fruit weight (NFR → FRW; β = 0.54) (**Figure 6A**). Together, these relationships describe a sequential reproductive pathway in which floral bud formation promotes flower production, flower production enhances fruit set, and fruit number ultimately determines fruit weight. The model explained 98% of the variance in NFL, 24% of the variance in NFR, and 29% of the variance in FRW.

Graphical inspection of the data revealed a curvilinear relationship between flower number and fruit number. Accordingly, a quadratic regression model was fitted. The model predicted a maximum fruit set of 8.35 fruits at approximately 17 flowers, after which additional increases in flower number were associated with a reduction in fruit number (**Figure 6B**).

#### 3.3.2 *Hylocereus costaricensis*– second production cycle

During the second production cycle, the reproductive path model showed a satisfactory global fit under robust estimation (χ² (2) = 1.62, p = 0.45; CFI = 1.00; TLI = 1.00; RMSEA = 0.00; SRMR = 0.03), sorting the suitability of the proposed structure to represent the relationships among the variables involved in the reproductive sequence, from floral buds to fruit weight.

Direct effects indicated that NFB positively influenced NFL (β = 0.81, p < 0.001), explaining 66% of its variance. In turn, NFL exerted a positive effect on FRW (β = 0.56, p < 0.001), accounting for 35% of its variability. Additionally, both NFB (β = 0.51, p < 0.001) and NFL (β = 0.27, p < 0.1) showed positive effects on NFR, jointly explaining 56% of its variance.

Complementing the direct pathways, **Table 6** summarizes the indirect and total effects estimated by the path model. The indirect pathway from floral bud number to fruit weight through flower number (NFB → NFL → FRW) was significant (β = 0.51, *P* < 0.01), indicating that part of the influence of floral bud production on fruit weight was mediated through flower production. In contrast, the indirect pathway from floral bud number to fruit number through flower number (NFB → NFL → NFR) was marginally significant (β = 0.22, *P* = 0.06), suggesting limited statistical evidence that flower production partially mediated the relationship between floral bud production and fruit number.

**Table 6:** Indirect and total effects of the reproductive pathway model on pitahaya yield during the second production cycle.

Effects	Estimation	EE	Z	p value
Indirect				
NFB ->NFL-> FRW	0.51	0.08	5.74	< 0.01
NFB -> NFL -> NFR	0.22	0.12	1.88	0.06
Total				
NFB -> NFR	0.73	0.09	7.97	< 0.01

The table presents the indirect and total effects of the number of floral buds (NFB) on fruit weight (FRW) and the number of fruits (NFR), with the number of flowers (NFL) included as the mediating variable. Standard errors (SE), Z statistics, and corresponding P values are also reported.

Por lo tanto, NFL actuó como una variable mediadora parcial en las relaciones de NFB con FRW y NFR, lo que significa que la producción de flores explicó parte de la transición de los botones florales hacía frutos y peso de fruto. No obstante, para la relación NFB → NFR, el efecto total estandarizado fue significativo (β = 0.73, p < 0.01), ya que integró tanto el efecto directo significativo de NFB sobre NFR (β = 0.51, p < 0.01) como el efecto indirecto marginalmente significativo mediado por NFL (β = 0.22, p = 0.06), lo que evidencia que la producción de botones florales constituye un componente relevante para explicar el número final de frutos, aunque parte de esta influencia se canaliza a través de la floración.

#### 3.3.3 *Hylocereus undatus* cv. Vietnam White– first production cycle

**Figure 7:**
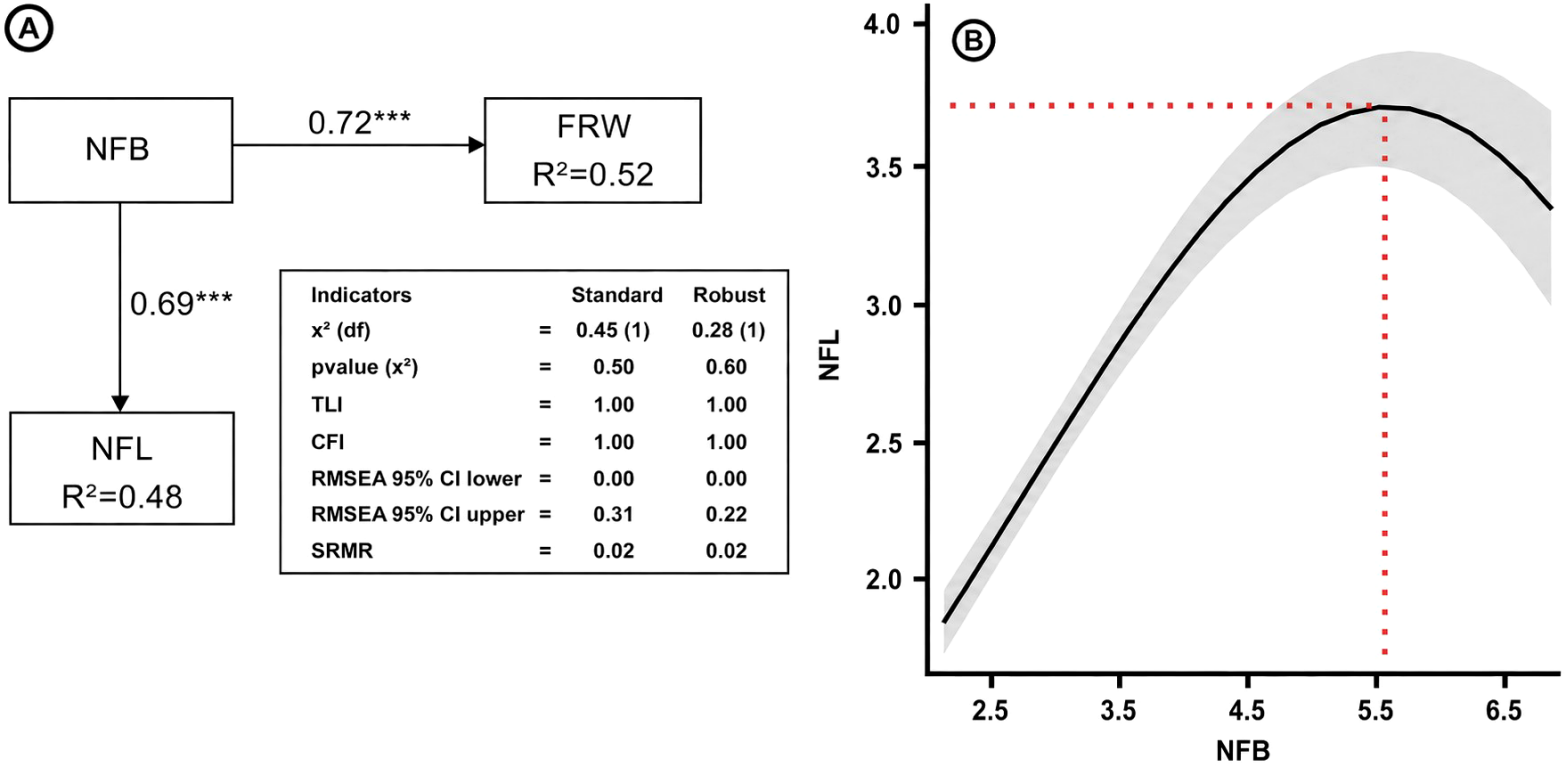
Reproductive path model of *H. undatus* cv. Vietnam White during the first production cycle: Direct effects and R^2^. **(A)** presents the reproductive path model for *H. undatus* cv. Vietnam White, including the overall goodness-of-fit indices, the standardized direct effects, and the estimated relationships among the number of floral buds (NFB), number of flowers (NFL), and fruit weight (FRW). Statistical significance is indicated by *** (*P* < 0.001). Coefficients of determination (R²) for the endogenous variables are also reported. **(B)** shows the fitted generalized Poisson regression model with second-order orthogonal polynomial terms describing the relationship between NFB and NFL, allowing identification of the floral bud number at which maximum flower production is predicted.

The reproductive path model showed an adequate global fit under robust (χ²(df) = 0.28(1), p = 0.60; CFI = 1.00; TLI = 1.00; RMSEA IC 95% = [0.00, 0.22]; SRMR = 0.02. In this model, NFR was excluded because it was perfectly correlated with NFL (r = 1), indicating that all recorded flowers successfully developed into fruits during the evaluated production cycle (Singh et al., 2026). In the final model structure, number of floral bud (NFB) exerted positive direct effects on both FRW (β = 0.72, p <.01) and NFL (β = 0.69, p <.01), explaining 52% and 48% of the variance, respectively (**Figure 7A**).

Graphical exploration of the data revealed a nonlinear relationship between flower number (NFL) and fruit number (NFR), prompting the fitting of a quadratic regression model. The results indicated that maximum fruit production (3.71 fruits) was achieved at approximately 5.5 flowers (**Figure 7B**). Beyond this threshold, further increases in flower number were associated with a decline in fruit production, suggesting a saturation effect in the reproductive capacity of the plant.

## 4 Discussion

### 4.1 Biotechnological synergy in the rhizosphere: biochar as an ecological niche for the plant growth-promoting activity of *Trichoderma viride*

The combined application of rice husk biochar and T. viride (T04) produced the highest cumulative fruit weight in *H. costaricensis* (4122.24 g vs. 2983.13–3103.07 g; **Table 7**) and was associated with the most favorable trends in floral bud and fruit production toward the end of the experimental period. (**Figure 3**). A key driver behind this synergistic response is likely the optimization of the rhizosphere microhabitat. The rhizosphere is a dynamic habitat where roots, soil particles, microorganisms, and root exudates interact to regulate nutrient cycling and plant physiology. Within this system, biochar enhances pore connectivity, water retention, and cation exchange capacity, creating favorable microsites for beneficial fungi such as *Trichoderma viride*. In turn, *T. viride* improves nutrient availability, reshapes the rhizosphere microbiome, and stimulates root growth. Collectively, these processes explain why T04 outperformed the single biochar or T. viride applications, as it established a biologically enriched rhizosphere that promoted resource acquisition and productivity (Woo et al., 2023; Contreras et al., 2024). Biochar produced at 710 °C by pyrolysis is characterized by a high specific surface area and cation exchange capacity (CEC) (Alkharabsheh et al., 2021; Kabir et al., 2023; Liu et al., 2025; Curi et al., 2026), which can function as a protective biological carrier that shields *T. viride* from competitive displacement and desiccation (Warnock et al., 2007; Lehmann and Joseph, 2015; Kabir et al., 2023). Under the alkaline soil conditions of Jaén (pH 7.6), where phosphorus (P) availability is frequently limited by calcium-mediated precipitation (Hinsinger, 2001; Marschner, 2012), the production of organic acids and siderophores by *Trichoderma* spp (Tyśkiewicz et al., 2022; Song et al., 2023). likely enhanced nutrient solubilization and uptake within this porous microhabitat.

**Table 7:** Fixed effects on the reproductive components *of H. costaricensis*: global analysis using generalized linear models (GLMs).

Factors/Treatments	NFB	NFL	NFR	FRW	SL
ANOVA (p-value)					
Blok	0.29	0.30	0.15	0.76	0.05
Biochar (BI)	0.03	0.02	0.15	< 0.01	0.06
*Trichoderma viride* (TV)	0.96	0.89	0.51	< 0.01	0.47
BI: TV	0.06	0.02	0.10	< 0.01	0.60
Means adjusted according to treatment					
T01	23.91ab	18.17ab	12.16	2983.13b	139.22
T02	24.48ab	18.20ab	11.93	3103.07b	140.60
T03	20.41b	14.86b	11.14	2958.29b	139.11
T04	27.82a	22.10a	14.05	4122.24a	139.89

The table presents the significance levels of the fixed effects, with block included as a control factor and biochar application (BI), Trichoderma viride inoculation (TV), and the BI × TV interaction included as explanatory factors. The evaluated response variables were the number of floral buds (NFB), number of flowers (NFL), number of fruits (NFR), fruit weight (FRW), and final stem length (SL). Adjusted means for each treatment are also presented for each response variable. Different lowercase letters within the same column indicate significant differences among treatments after false discovery rate (FDR) correction (P < 0.05). Treatments were defined as follows: T01, no biochar and no T. viride; T02, biochar and no T. viride; T03, no biochar and T. viride; and T04, biochar and T. viride.

**Figure 8:**
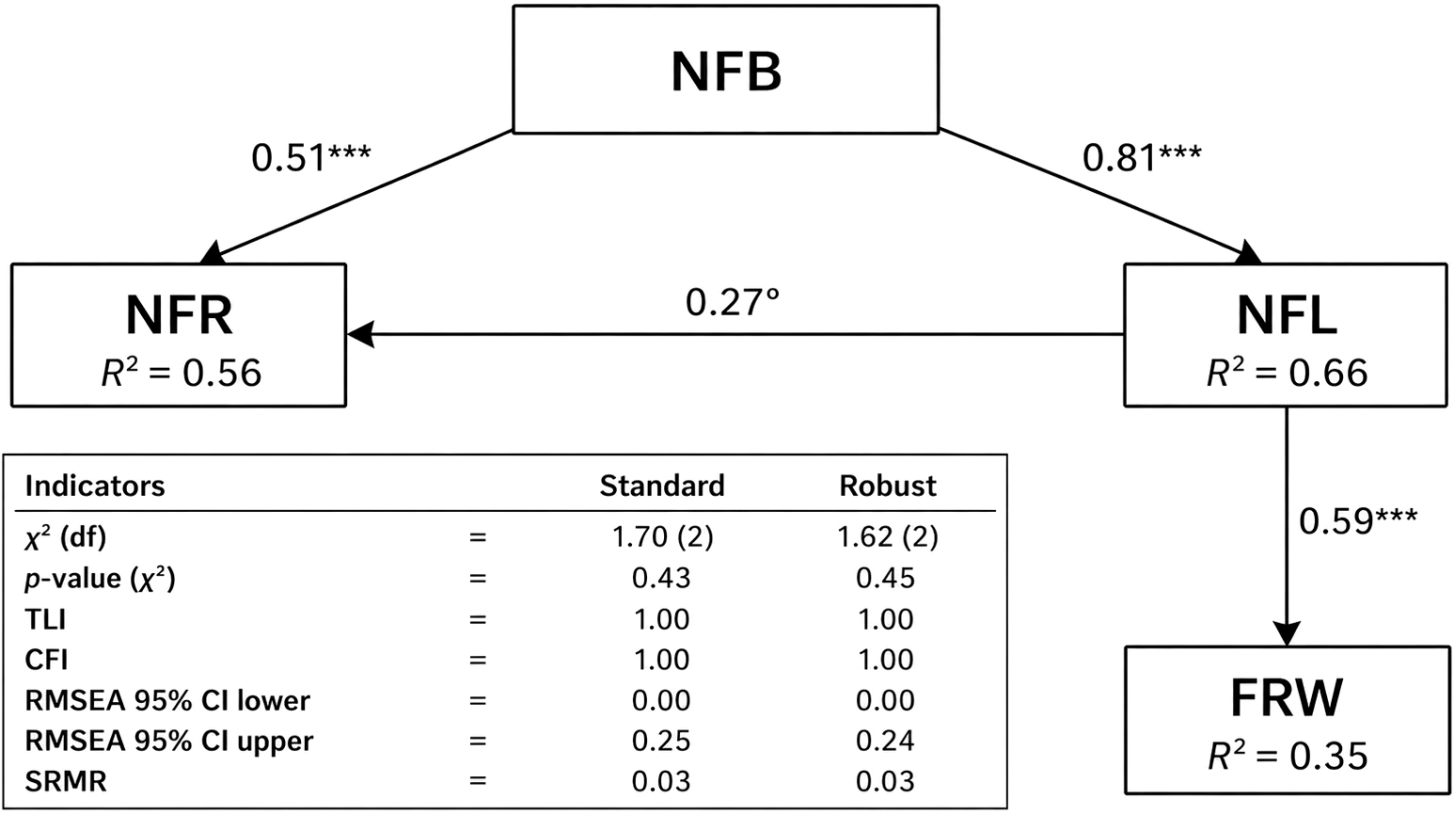
Reproductive path model of *H. costaricensis* during the second production cycle: direct effects and R^2^. The path model shows the standardized direct effects, coefficients of determination (R²), and overall goodness-of-fit indices. The variables included were the number of floral buds (NFB), number of flowers (NFL), number of fruits (NFR), and fruit weight (FRW). Direct effects are represented by arrows (→), and significance levels are indicated as follows: ° *P* < 0.1, and *** *P* < 0.001.

Interestingly, the most consistent effect of the T04 treatment was observed on individual fruit weight (p < 0.01) rather than on total fruit number, which showed only a non-significant positive trend (**Table 7**). This pattern strongly suggests a whole-plant physiological modulation of fruit growth and biomass accumulation rather than an increase in initial fruit set. From a source-sink perspective, this interpretation is mathematically sorted by our path analysis; in the first production cycle, a powerful direct effect was observed from fruit number to final fruit weight (*β* = 0.54, *p* < 0.001), while in the second cycle, flower number acted as a primary mediator of fruit weight (*β* = 0.59, *p* < 0.001; **Figures 6**, **8**). Although biochar applications have been reported to lower stress-induced abscisic acid levels (Ekinci et al., 2022; Lee et al., 2025), and *Trichoderma* spp. are known to stimulate endogenous auxin production (Harman et al., 2004; Cui et al., 2025), auxin regulates reproductive development by coordinating cell division, cell expansion, vascular differentiation, and photoassimilate allocation toward developing reproductive organs. During fruit development, elevated auxin concentrations stimulate sink establishment within young ovaries by enhancing the expression of sugar transporters, invertases, and genes involved in cell-wall loosening, thereby increasing assimilate import and sustaining fruit growth. In addition, auxin interacts synergistically with gibberellins and cytokinins to delay premature abscission and maintain reproductive sink activity, ultimately improving fruit size rather than necessarily increasing fruit number. These physiological mechanisms are consistent with the higher individual fruit weight observed in the combined biochar–*Trichoderma* treatment (Woo et al., 2023; Contreras et al., 2024). Our model-driven results demonstrate that the primary agronomic expression of the T04 synergy operates by strengthening sink activity in developing ovaries. From a source–sink perspective, mature cladodes function as the principal carbon sources through photosynthetic assimilation and temporary carbohydrate storage, whereas developing flowers and fruits constitute strong metabolic sinks. Sink strength depends not only on sink size but also on its capacity to import and metabolize photoassimilates. During reproductive development, sucrose transported via the phloem is hydrolyzed by cell-wall invertases into hexoses, generating concentration gradients that facilitate continuous carbon import into developing fruits. Concurrently, hormonal signaling—particularly auxin and gibberellins—maintains vascular connectivity and phloem unloading efficiency, thereby reinforcing assimilate translocation toward reproductive tissues. Treatments that improve nutrient acquisition and photosynthetic efficiency, such as the combined application of biochar and *Trichoderma*, are therefore expected to strengthen sink activity, resulting in greater biomass accumulation per fruit without necessarily increasing fruit number. This enhanced sink activity allows fruits to accumulate more biomass during cell expansion, improving resource‑use efficiency without a proportional increase in fruit number.

Beyond hormonal modulation, *Trichoderma* activates multiple biochemical pathways associated with plant metabolism. Root colonization induces antioxidant enzymes including superoxide dismutase, catalase, ascorbate peroxidase, and peroxidases, which reduce oxidative stress while preserving photosynthetic performance. Simultaneously, enhanced activities of nitrate reductase, glutamine synthetase, sucrose synthase, and cell-wall invertases improve nitrogen assimilation and carbon partitioning toward actively growing tissues. These coordinated responses increase primary metabolism by promoting amino acid, carbohydrate, and protein biosynthesis, while also stimulating secondary metabolic pathways involved in phenylpropanoid synthesis and stress protection. Collectively, these biochemical adjustments enhance resource-use efficiency and sort sustained reproductive development under field conditions.

This treatment divergence became most pronounced during the final stages of the experiment (M05–M06; **Figure 3**), indicating a gradual, cumulative establishment of both the biochar matrix and the fungal population within the rhizosphere (Lehmann et al., 2011). Although we did not directly quantify microbial rhizosphere colonization, specific enzymatic activities (such as ACC deaminase), or internal plant hormone dynamics, our agronomic models provide a robust whole-plant level validation of these interactions. Future physiological studies incorporating post-treatment soil biochemical properties, targeted hormonal profiling, and source-sink balance manipulations are warranted to fully map the precise molecular pathways underlying these rhizospheric interactions.

### 4.2 Carrying capacity and reproductive self-regulation: deciphering nonlinearity in cactus productivity

The reproductive path analysis revealed that flowering acted as a key mediating variable between floral buds and final fruit weight in *H. costaricensis* (**Figure 6**). This finding demonstrates that the effect of the T04 treatment was not solely attributable to enhanced initial floral induction, but rather to an optimization of the metabolic transition efficiency along the bud–flower–fruit continuum. Previous studies have established that such reproductive efficiency is strictly regulated by assimilate availability and competing demands among synchronous reproductive sinks, for example cucumber, pepper and olive (Marcelis, 1996; Marcelis et al., 2004; Rosati et al., 2023).

A particularly relevant finding of our model was the identification of a non-linear, quadratic relationship between flowering and fruit production in *H. costaricensis* during the first growing season, revealing a clear saturation threshold at approximately 17 flowers per plant (**Figure 6B**). Beyond this critical carrying capacity, further increases in flower number failed to translate into higher fruit set, mathematically illustrating the occurrence of regulated physiological abortion processes similar to those documented in olive, almond, and other perennial species (Cunningham et al., 2020; Chiamolera et al., 2023; Rosati et al., 2023). Such reproductive constraints are ubiquitous in perennial fruit crops, where frequently fewer than 10% of flowers successfully develop into mature fruits (Agustí et al., 1982; Agustí et al., 2025), dropping to less than 1% in highly taxing species like avocado (Sedgley, 1980; Garner and Lovatt, 2016; Alcaraz and Hormaza, 2021).

In the genus Hylocereus, where episodic flower bud emission typically far exceeds the plant’s structural and metabolic capacity to sustain heavy, fleshy fruits, the activation of internal self-regulation mechanisms is evolutionary expected. From a physiological standpoint, this bottleneck is primarily driven by localized competition for non-structural carbohydrates (Marcelis et al., 2004; Agustí et al., 2025). While the precise molecular triggers were not directly quantified in this study, established literature indicates that carbohydrate starvation within the ovary can act as a primary signal that modulates downstream hormone-mediated pathways (such as auxin depletion and increased abscisic acid/ethylene ratios), which ultimately activate the enzymatic degradation of the cell wall via pectinases and cellulases within the abscission zone (Marcelis, 1996; Al-Dulaimy et al., 2023; Qiu et al., 2026).

This interpretation is sorted by several complementary processes rather than by a single physiological mechanism. First, biochar improves soil physical properties by increasing water retention, aeration, and cation exchange capacity, thereby enhancing nutrient availability in the rhizosphere. Second, *Trichoderma* promotes root development, nutrient solubilization, and phytohormone-mediated signaling, increasing nutrient uptake efficiency. Third, improved nutritional status enhances photosynthetic carbon assimilation and carbohydrate accumulation, which ultimately sorts stronger reproductive sinks. Together, these interacting processes provide a biologically coherent explanation for the superior fruit biomass observed under the combined treatment, while acknowledging that the specific contribution of each mechanism was not directly quantified in the present study.

Interestingly, this quadratic saturation pattern was not evident during the second campaign (**Figure 8**), suggesting a cumulative, long-term benefit of the biochar–*Trichoderma* synergy (T04). We hypothesize that over time, this rhizospheric interaction progressively enhanced the overall nutritional and metabolic status of the structural cladodes. By upgrading the baseline carbon storage of the host plant, the treatment effectively raised the carrying capacity threshold, allowing a greater proportion of flowers to complete fruit conversion successfully without triggering the energetic self-regulation threshold. increasing carbon availability for reproductive development and thereby raising the plant’s reproductive carrying capacity. Similar mechanisms have been reported in *Prunus armeniaca* L., where carbohydrate metabolism and endogenous hormonal balance are key determinants of pistil survival, flower retention, and successful fruit set. In particular, impaired carbohydrate metabolism and hormonal imbalances have been associated with reproductive organ abortion during early flower development (Zhang and Wei, 2022; Zhao et al., 2022), Although these mechanisms were not directly evaluated in the present study, they provide a plausible physiological explanation for the improved reproductive performance observed under the combined biochar–*Trichoderma* treatment (Zhang and Wei, 2022; Zhao et al., 2022).

The beneficial effects observed under the combined biochar–Trichoderma treatment is likely associated with the progressive establishment of Trichoderma in the rhizosphere. Sustained root colonization has been reported to enhance nutrient acquisition, stimulate root development, modulate phytohormone signaling, and improve overall plant physiological performance, thereby generating cumulative benefits throughout crop development (Poveda, 2021).

This dynamic contrasted sharply with *H. undatus* cv. Vietnam White, where the flower–fruit relationship remained predominantly linear (**Figure 8**). This linear behavior is likely attributed to its high self-compatibility and superior inherent fruit-set efficiency under the study conditions (Pérez et al., 2022; Karunakaran et al., 2026), meaning it had not yet reached its physiological saturation point during the evaluation period. Consequently, our model underscores that flowering in *H. costaricensis* functions as a critical, non-linear mediator of reproductive success. While direct quantification of internal phytohormones and abscission-related enzymatic profiles remains an essential step for future research, these path analysis results provide a solid functional framework highlighting that maximizing yield in red pitahaya depends on managing flower-to-fruit conversion efficiency rather than merely forcing floral induction.

### 4.3 Implications for differentiated management: toward precision agriculture based on genotype and bioinput profiles

Taken together, these findings sort a precision-management framework tailored to the distinct reproductive behaviors and carrying capacities of each pitahaya genotype. The T04 treatment (biochar + TV) appears to have optimized rhizosphere nutrient availability and hormonal balances, but its agronomic expression is highly genotype dependent, reinforcing the premise that bioinput efficiency is strictly modulated by the host’s genetic background and developmental stage (Patwary et al., 2013; Cardoso et al., 2024). For *H. costaricensis*, which exhibits a non-linear reproductive transition and a stark saturation threshold (~17 flowers per plant), management strategies should focus on raising this physiological ceiling. In this scenario, the cumulative biochar- *Trichoderma* synergy acts as a metabolic buffer (Haider et al., 2021).

From a metabolic perspective, the observed response may involve the coordinated regulation of both primary and secondary metabolism. Enhanced nutrient availability and root colonization by *Trichoderma viride* are expected to stimulate primary metabolic pathways associated with carbon and nitrogen assimilation, including photosynthetic carbon fixation, sucrose metabolism, amino acid biosynthesis, and protein synthesis. These processes increase the production of photoassimilates that sustain vegetative growth and reproductive sink demand. Simultaneously, secondary metabolism may also be reinforced through activation of the phenylpropanoid pathway and the biosynthesis of antioxidant compounds, flavonoids, and other specialized metabolites involved in oxidative stress mitigation and cellular protection. The integration of these metabolic processes improves physiological performance by maintaining photosynthetic activity, reducing oxidative damage, and increasing carbon-use efficiency during fruit development, thereby contributing to higher reproductive biomass under the combined biochar–*Trichoderma* treatment.

By enhancing nutrient solubilization and potentially modulating the carbohydrate-to-phytohormone signaling balances, this bioinput combination strengthens resource translocation toward the reproductive sinks (López et al., 2015; Vinci et al., 2018; Singh et al., 2026). Mechanistically, the highly porous structure of rice husk biochar improves soil water-holding capacity and cation exchange capacity while creating a favorable physical niche for *Trichoderma viride* colonization (Liu et al., 2017; Hu et al., 2025). Once established, *T. viride* stimulates lateral root branching and elongation through the production of organic acids and auxin-like compounds, thereby increasing the effective absorptive surface of the root system (Garstecka et al., 2023). Improved nutrient and water acquisition enhances photosynthetic performance within the photosynthetically active cladodes, the primary source organs in pitahaya (Niechayev et al., 2023). Consequently, greater carbon assimilation promotes the accumulation of non-structural carbohydrates (starch and soluble sugars) before anthesis, providing the metabolic reserves required to sustain flower development, fruit set, and subsequent fruit growth through more efficient assimilate partitioning toward reproductive sinks (Li et al., 2018; Rossouw et al., 2024).

This allows the plant to sustain a heavier single-fruit weight (**Table 7**) without triggering the carbon-deprivation pathways that normally activate premature abscission under high sink densities (Botton and Ruperti, 2019). This physiological advantage is likely associated with the maintenance of carbohydrate availability during the critical stages of ovary and early fruit development, when the balance between carbon sly and hormonal signaling determines organ retention. Under carbohydrate-limited conditions, a sharp decline in non-structural carbohydrate concentrations within the developing ovary disrupts polar auxin transport, which normally maintains cells within the abscission zone (AZ) in an ethylene-insensitive state (Larson et al., 2022). Reduced auxin flux increases the sensitivity of AZ cells to endogenous ethylene and abscisic acid (ABA), whose biosynthesis is enhanced under carbon starvation (Yuan et al., 2025). This hormonal imbalance activates a transcriptional cascade that induces the *de novo* synthesis and secretion of cell wall-degrading enzymes, including polygalacturonases (pectinases) and endo-β-1,4-glucanases (cellulases), into the apoplast. These enzymes hydrolyze the middle lamella and the cellulose matrix, progressively weakening cell-to-cell adhesion and ultimately causing premature organ abscission. By maintaining carbohydrate availability above this critical threshold, the combined application of rice husk biochar and *Trichoderma viride* likely prevents activation of this signaling cascade, thereby improving fruit retention and enabling greater assimilate allocation to the remaining fruits (Li et al., 2015). Consequently, for this cultivar, biostimulation should target the expansion of the sink-holding capacity during peak flowering events.

Conversely, *H. undatus* cv. Vietnam White exhibited a predominantly linear flower-to-fruit progression under our experimental conditions. Rather than indicating an unlimited productive capacity, this linearity reflects a lower baseline of floral emission driven by its extended juvenile phase and delayed reproductive development in the studied region (**Table 7**; **Figure 8**). Because this genotype has not yet reached its physiological saturation threshold, its high self-compatibility ensures that the few flowers produced are efficiently converted into fruits (Pérez et al., 2022; Jadhav et al., 2025). Therefore, precision management for *H. undatus* should not merely force premature floral induction—which could deplete non-structural carbohydrates in immature cladodes—but should instead prioritize agronomic inputs (such as targeted biochar applications) that accelerate vegetative structural establishment and biomass accumulation. Once a robust structural framework is achieved, targeted biostimulation can safely exploit its linear fruit-set potential. This genotype-specific approach aligns with contemporary precision agriculture frameworks, which emphasize tailoring management inputs to the specific biological constraints and productive potential of distinct crop phenotypes to optimize resource-use efficiency and long-term yield sustainability (Panda et al., 2010; Longchamps et al., 2022).

## 5 Conclusions

The reproductive response of pitahaya was highly dependent on both the cultivar and the evaluation period, demonstrating that bioinput effects are context-specific. In *Hylocereus costaricensis*, treatment-induced benefits extended across the entire reproductive sequence—particularly during the late evaluation stages—whereas the response in *H. undatus* cv. Vietnam White was more restricted and linear. The combined application of biochar and *Trichoderma viride* (T04) exhibited the most consistent performance, driving a substantial increase in final reproductive biomass for *H. costaricensis* compared to the remaining treatments, alongside positive trends in floral bud and fruit counts. These findings indicate that the synergistic bioinput combination does not merely stimulate isolated components but rather enhances transition efficiencies along the reproductive continuum to improve overall yield.

A central highlight of this study was the identification of a non-linear relationship between flowering and fruit set in H. costaricensis during the first production cycle, which revealed an intrinsic reproductive carrying capacity threshold capped at approximately 17 flowers per plant. Beyond this saturation point, further flowering did not translate into higher fruit numbers. During the second production cycle, reproductive success in H. costaricensis was dictated by a significant indirect effect of floral buds on final fruit weight mediated through flower number. Consequently, precision management strategies for this genotype should prioritize effective flowering and fruit filling over the induction of massive, unsustainable floral bud emissions.

Finally, the divergent path models confirm that the key determinants of yield are cultivar-specific. Optimizing yield in *H. costaricensis* requires a holistic management of the entire sequential pathway (floral buds → flowers → fruits → fruit weight) to raise its physiological carrying capacity. Conversely, for *H. undatus* cv. Vietnam White, the focus should prioritize accelerating vegetative structural establishment and cladode biomass accumulation during its extended juvenile phase; once this robust framework is achieved, management can safely exploit its high inherent fruit-set capacity and linear flower-to-fruit progression. Because direct quantifications of phytohormones or ACC deaminase activity were not performed, the physiological mechanisms proposed herein represent a functional baseline that warrants further validation through future metabolic profiling, hormonal analyses, and multi-location field trials.

## Data Availability

The original contributions presented in the study are included in the article/**Supplementary Material.** Further inquiries can be directed to the corresponding author.
