# Supplementary material for: Path analysis of the efficiency in the bud-flower-fruit transition of pitahaya: the biochar-Trichoderma viride combination improves yield in Hylocereus costaricensis and H. undatus cv. Vietnam White
Source: Front Plant Sci. 2026 Jul 31;17:1901632. doi: 10.3389/fpls.2026.1901632 (PMC13474990; doi:10.3389/fpls.2026.1901632)
Supplement: Supplementary file 1 [file SupplementaryFile1.docx]

# *Supplementary Material*

## 1. Supplementary Figures

## Supplementary Tables

**Figure S1.** Monthly meteorological parameters recorded during the first and second growing seasons of the pitahaya.

**Figure S2.** Pitahaya Planting and Irrigation System

## 2. Supplementary Tables

**Table S1.** Characteristics of rice husk biochar

| **Parámetro** | **Unidad** | **Valor** |
| --- | --- | --- |
| pH (1:20 H_2_O) | - | 7.3 |
| CE (1:20 H_2_O) | dS m⁻¹ | 1.061 |
| WHC | g H_2_O g^-1^ biochar | 1.70 |
| Corg | g kg^-1^ | 383.46 |
| Nt | mg g^-1^ | 4.93 |
| Zn | mg kg⁻¹ | 52.01 |
| Cd | mg kg⁻¹ | 0.51 |
| Mg | mg kg⁻¹ | 33.93 |
| Fe | mg kg⁻¹ | 8.12 |
| Co | mg kg⁻¹ | 0.45 |
| Mn | mg kg⁻¹ | 30.80 |
| AL | mg kg⁻¹ | 21.07 |
| Pb | mg kg⁻¹ | 52.01 |
| Cr | mg kg⁻¹ | 1.01 |
| Na | mg kg⁻¹ | 412.98 |
| Ca | mg kg⁻¹ | 14408 |
| K | mg kg⁻¹ | 7052 |
| WHC: water high capacity | | |

**Table S2.** Model specifications and AIC by response variable

| Variables | Distribution | link | AIC | Dispersion |
| --- | --- | --- | --- | --- |
| *Hylocereus costaricensis*  Longitudinal evaluation | | | | |
| NFB | Genpois | Logarithmic | 1437 | Time |
| NFL | Genpois | Logarithmic | 1290 | Time |
| NFR | Tweedie | Logarithmic | 1003 | Time |
| FRW | Gamma | Logarithmic | 3803 | Time |
|  | Binomial | Logit | 210 |  |
| SL | Tweedie | Logarithmic | 3163 | Time + TV |
| Global assessment | | | | |
| NFB | Genpois | Logarithmic | 505 | - |
| NFL | Genpois | Logarithmic | 480 | - |
| NFR | Genpois | Logarithmic | 422 | - |
| FRW | Lognormal | Logarithmic | 1152 | - |
| SL | Gamma | Logarithmic | 346 | - |
| Variety Vietnam White  *Hylocereus undatus cv. Vietnam White* | | | | |
| NFB | Binomial | Logit | 206 | - |
| NFL | Binomial | Logit | 188 | - |
| NFR | Binomial | Logit | 207 | - |
| SL | Tweedie | Logarithmic | 3683 | Time |
| Global assessment | | | | |
| NFB | C-M-P | Logarithmic | 230 | - |
| NFL | C-M-P | Logarithmic | 156 | - |
| NFR | C-M-P | Logarithmic | 156 | - |
| FRW | Binomial | Logit | 107 | - |
|  | Gamma | Logarithmic | 594 | - |
| SL | Gamma | Logarithmic | 346 | - |
| *Note*. The table presents the distributions used (Tweedie, Gamma, Binomial, GenPois: Generalized Poisson, and C–M–P: Conway–Maxwell–Poisson) together with their corresponding link functions (log and logit), as well as the AIC values of the selected models and the variables included to model dispersion, when applicable. The analyzed variables were number of floral buds (NFB), number of flowers (NFL), number of fruits (NFR), stem length (SL), and fruit weight (FRW). In the case of FRW, due to the high frequency of zeros, the variable was decomposed into two components for the implementation of a hurdle model: (ia binary component modeling the absence/presence of production, and (ii) a positive component considering only experimental units containing at least one productive plant. This approach was applied both in the overall evaluation of *Hylocereus undatus* cv. Vietnam White and in the longitudinal evaluation of *Hylocereus costaricensis*. | | | | |

**Table S3.** Effect of time and inoculation with *Trichoderma viride* on the dispersal of pitahaya reproductive parts.

|  | *Hylocereus costaricensis* | | | | | *Hylocereus undatus cv. Vietnam White* |
| --- | --- | --- | --- | --- | --- | --- |
|  | NFB | NFL | NFR | FRW | SL | SL |
| Time | < 0.01 | < 0.01 | < 0.01 | < 0.01 | < 0.01 | < 0.01 |
| M01 | 5.71a | 5.77a | - | - | 3.29a | 2.47a |
| M02 | 8.54a | 7.14a | 1.52a | 3.42c | 1.89b | 2.13a |
| M03 | 1.08b | 1.3b | 1.55a | 7.24b | 1.00c | 1.65ab |
| M04 | 0.35c | 0.49c | 1.00b | 3.13c | 0.26d | 1.03b |
| M05 | 0.68bc | 0.12d | 0.06c | 7.28b | 0.02e | 0.47c |
| M06 | - | - | - | 91.09a | 0.00f | 0.01d |
| M07 | - | - | - | - | - | 0.00e |
| *T. viride* | - | - | - | - | < 0.01 | - |
| Con | - | - | - | - | 0.27a | - |
| Sin | - | - | - | - | 0.12b | - |
| *Note*. In *Hylocereus costaricensis*, the table shows the effect of the time on the dispersión of theb number of floral buds (NFB), number of flowers (NFL), number of fruits (NFR), fruit weight (FRW), and stem length (SL), as well as the effect of *Trichoderma viride* inoculation on the dispersion of SL. In *Hylocereus undatus* cv. Vietnam White, only the effect of time on the dispersion of SL is presented. Additionally, comparisons of dispersion among evaluation times and among *T. viride* inoculation levels are shown according to the analyzed variable. Different letters among times or inoculation levels indicate significant differences (p < 0.05; correction FDR). M01–M07 correspond to the evaluation dates described in Table 2. | | | | | | |
